# SATB1 as oncogenic driver and potential therapeutic target in head & neck squamous cell carcinoma (HNSCC)

**DOI:** 10.1038/s41598-020-65077-y

**Published:** 2020-05-25

**Authors:** Omkar Panchal, Gunnar Wichmann, Reidar Grenman, Lisa Eckhardt, Leoni A. Kunz-Schughart, Heike Franke, Andreas Dietz, Achim Aigner

**Affiliations:** 1 Rudolf-Boehm-Institute for Pharmacology and Toxicology, Clinical Pharmacology, University of Leipzig, Leipzig, Germany; 2 Dept. of Otolaryngology, Head and Neck Surgery, University Clinic Leipzig, Leipzig, Germany; 3 Dept. of Otorhinolaryngology-Head and Neck Surgery, Turku University and Turku University Hospital, Turku, Finland; 4 OncoRay – National Center for Radiation Research in Oncology, Faculty of Medicine and University Hospital Carl Gustav Carus, TU Dresden, and Helmholtz-Zentrum Dresden, Rossendorf, Germany; 5 National Center for Tumor Diseases (NCT), partner site Dresden, Dresden, Germany; 6 Rudolf-Boehm-Institute for Pharmacology and Toxicology, University of Leipzig, Leipzig, Germany

**Keywords:** Oral cancer, Oncogenes

## Abstract

The Special AT-rich sequence binding protein 1 (SATB1) is a genome organizer protein that controls gene expression of numerous genes by regulating chromatin architecture and targeting chromatin-remodeling/-modifying enzymes onto specific chromatin regions. SATB1 is overexpressed in various tumors. In head and neck squamous cell carcinoma (HNSCC), SATB1 upregulation is correlated with TNM classification, metastasis, poor prognosis and reduced overall survival. In this paper, we comprehensively analyze cellular and molecular effects of SATB1 in a large set of primary cell lines from primary HNSCC or metastases, using RNAi-mediated knockdown *in vitro* and, therapeutically, in tumor xenograft mouse models *in vivo*. In a series of 15 cell lines, major differences in SATB1 levels are observed. In various 2-D and 3-D assays, growth inhibition upon efficient siRNA-mediated SATB1 knockdown depends on the cell line rather than initial SATB1 levels. Inhibitory effects are found to be based on cell cycle deceleration, apoptosis induction, decreased HER3 and Heregulin A&B expression, and effects on EMT genes. *In vivo*, systemic treatment of tumor xenograft-bearing mice with siRNAs formulated in polymeric nanoparticles inhibits tumor growth of two HNSCC xenograft models, resulting from therapeutic SATB1 reduction and concomitant decrease of proliferation and induction of apoptosis. In conclusion, SATB1 represents a promising target in HNSCC, affecting crucial cellular processes and molecular pathways.

## Introduction

Worldwide, incidence rates of head and neck squamous cell carcinoma (HNSCC) are ~ 600,000 new cases per year and increasing, and they mostly present as locally advanced disease^1^. Major etiologies of HNSCCs are tobacco and/or alcohol and viral infections like HPV (human papilloma virus; type 16 and 18) or Epstein-Barr virus (EBV). Genetic predisposition, reduced immunity, exposure to radiation, chewing betel nut or carcinogens contribute to the development and progression of HNSCC as well^2–5^. Prognosis is generally poor with little improvement towards increasing patients’ overall survival (OS) and quality of life; hence, novel therapeutic strategies are desperately needed.

The Special AT-rich sequence binding protein family consists of two proteins, SATB1 and SATB2, sharing 61% homology in their amino acid sequence^6^. SATB1 is a genome organizer protein that controls gene expression of numerous genes^7^. More specifically, SATB1 regulates chromatin architecture and gene regulation by targeting chromatin-remodeling and -modifying enzymes onto specific chromatin regions, thus acting as a ‘landing platform’^8^, and by folding chromatin into loops and changing the epigenetic status of chromatin in a region-specific manner^7^.

SATB1 is critical during embryonic development^9,10^ and plays a role in the development and healthy functioning of the immune system^11–13^. In adults, different physiological functions have been described, including its role in the maintenance of pluripotency of hematopoietic stem cells in the bone marrow^14^. SATB1 has been found to be upregulated in various solid tumors including HNSCC, and its role in cancer progression has thus been studied in different tumor entities in great detail (^15^; see^16,17^ for review). On the other hand, the analysis of TCGA (The Cancer Genome Atlas) expression data via UALCAN^18^ does not support SATB1 upregulation in solid tumors including HNSCC. Taken together, the role of SATB1 in HNSCC remains ambiguous due to limited and inconsistent data.

A series of publications show SATB1 upregulation in HNSCC as compared to normal tissue and a positive correlation with TNM classification, metastasis formation, poor prognosis and reduced OS^19–21^, while TCGA data do not support this notion (see above). Upon SATB1 knockdown, a reduction of EMT markers such as N-cadherin and beta catenin was reported, while E-cadherin was slightly elevated^20^. The correlation of EBV infection and increased SATB1 level during dysplasia was proven by immunohistochemical staining of nasopharyngeal tumor cells^22,23^. Functional studies using an HNSCC cell line with stable shRNA-mediated SATB1 knockdown further indicate that SATB1 promotes tumor growth, invasiveness and metastasis^20,24^. In contrast, other investigators found SATB2, rather than SATB1, to be the major player, and also reported undetectable SATB1 mRNA levels in HNSCC tissue samples^25^. Nonetheless, SATB1 (over-) expression is still considered as putative prognostic marker in HNSCC and may also be a promising therapeutic target.

Our study was designed to comprehensively and systematically analyze the cellular and molecular role of SATB1 in HNSCC by using transient RNAi-mediated knockdown *in vitro* and in tumor xenograft mouse models *in vivo*. A considerable range of cell lines from primary HNSCC and/or its metastasis were employed.

## Materials and Methods

All methods were carried out in accordance with relevant guidelines and regulations, as detailed below. Where applicable, experimental protocols were approved by an institutional and/or licensing committee as detailed below.

### Cell lines and culture conditions

Primary squamous carcinoma cell lines (University of Turku-Squamous Cell Carcinoma (UT-SCC)) have been established previously in the Grenman lab^26^ and FaDu cells were from ATCC/LGC (Wesel, Germany). All cell lines were newly authenticated using STR profiling or obtained from the original source within the last four years. Cells were cultured under standard conditions (37 °C, 5% CO_2_) in Dulbecco’s Modified Eagle’s Medium (DMEM) with Sodium Pyruvate (Sigma, Taufkirchen, Germany), supplemented with 10% (v/v) fetal calf serum and glutamine (both from Biochrom, Berlin, Germany).

### RNA preparation and mRNA detection by qPCR

RNA isolation was done by Extrazol (Blirt, Gdansk, Poland), following the manufacturer’s protocol. RNA amounts were determined spectrophotometrically. The RevertAid™ H Minus First Strand cDNA Synthesis Kit (Fermentas, St. Leon-Roth, Germany) was used to reversely transcribe 1 µg total RNA with random hexamer primers. The 1:10 diluted cDNA was used as template for the quantitative PCR performed at a final volume of 10 µl containing 1X gene expression master mix, 1X specific primers (see Suppl. Table 2) and the detection probe. The StepOnePlus™ Real Time System and PerfeCTa^®^ SYBR^®^ Green FastMix^®^, ROX (QuantaBio, Hilden, Germany) was employed according to the manufacturer’s instructions. Conditions were as follows: 15 sec. 95 °C, then 45 cycles comprising 95 °C for 10 sec, 55 °C for 10 sec and 72 °C for 10 sec. Levels of mRNA were determined by using the ∆∆Ct method based on the formula 2^-(CP(target)-CP(hk))^ with CP = cycle number at the crossing point. RPLP0 or actin was used as reference genes.

### Western Blotting, Immunocytochemistry and Immunohistochemistry

Immunodetection of proteins in cell/tissue lysates, fixed cells and paraffin sections was performed as described in the Suppl. Materials and Methods. Normal mucosa tissue samples for Western blots were obtained from chronic tonsillitis patients. This investigational study was conducted according the Declaration of Helsinki as approved by the ethics committee of the Medical Faculty of the University of Leipzig (votes no. 201–10–12072010 and no. 202–10–1207210). After receiving informed consent of the patients to participate in the study, samples were taken during surgical procedures under general anaesthesia and stored at -80 °C. Samples from patients who had not received any radiation or chemotherapy before sample collection were eligible for biomarker analyses done according to the approved protocols only after the completion of routine histopathological diagnosis confirming absence of any signs of malignancy.

### Heregulin ELISA

For heregulin beta-1 measurement, 600 µl cell supernatant from 12 well plates were collected at 72 h post transfection and centrifuged at 5000 rpm at 4 °C for 10 min, prior to storage at – 80 °C. Except for the blocking step, the manufacturer’s protocol (#900 K316, Peprotech, Hamburg) was followed. Plates were blocked with 5% BSA and 10% FCS (the same batch that was used for culturing and transfection experiment of the cells). Standards were run in parallel, yielding a linear equation which was used for determining Heregulin beta-1 protein levels in the samples.

### Cell transfection, anchorage-dependent proliferation, colony formation and spheroid assay

Cells were seeded in 12 well plates at different cell densities (15,000-40,000 cells/well), depending on the growth rate of each cell culture, one day prior to transfection with 5 nM siRNA in 1100 µl media using INTERFERin (Polyplus, Illkirch, France). In 96-well plates, the same siRNA concentration in 260 µl was used. At the time point of harvesting after 72 h or 96 h (120 h for Western blot), cell confluency was roughly 80-90%. For quantitation of anchorage-dependent proliferation, cells were harvested by trypsinization and counted by flow cytometry using an Attune® Acoustic Focusing Cytometer (Life Technologies, Darmstadt, Germany). The live cell population was selected based on the forward and sideward scatter. Colony formation and spheroid assays were done as described in the Suppl. Materials and Methods. For cell proliferation assays in the presence of caspase-3 inhibitor, see Suppl. Materials and Methods.

### Cell cycle analysis and apoptosis assays

For cell cycle analysis, cells were seeded into 12-well plates and transfected as described above. 72 h after transfection, 25-50 ng/ml nocodazole (Merck-Calbiochem, Darmstadt, Germany) was added for 12-18 hours to induce a G2/M arrest. After harvesting by trypsinization, cells were washed with PBS and fixed in 70% ethanol at 4 °C overnight. Fixed cells were incubated with 50 µg/ml RNase A for 30 min at 37 °C prior to the addition of 50 µg/ml propidium iodide for flow cytometric DNA analysis with an Attune® Acoustic Focusing Cytometer.

Apoptosis was determined by detection of active Caspase-3/7 using a bioluminescent Caspase-3/7 Glo® assay (Promega, Mannheim, Germany), as described in the Suppl. Materials and Methods.

### Mouse xenograft therapy study

Animal studies were performed in accordance with the national regulations and guidelines, and approved by the local authorities (Landesdirektion Sachsen, Germany). From seven primary cell lines selected for detailed analysis, only UT-SCC-42B and UT-SCC-14 cells formed subcutaneous tumors. After shaving the injection region, 5×10^6^ cells in 150 µl PBS were injected into both flanks of 6-8 week old immunodeficient mice (NOD/SCID/IL2r gamma (null), Jackson Laboratory, Bar Habor, ME) which were kept in cages with rodent chow (ssniff, Soest, Germany) and water available *ad libitum*. When solid tumors were established, mice were randomized into treatment and control groups. Polymeric nanoparticles (PEI/siRNA complexes) based on low molecular weight branched polyethylenimine PEI F25-LMW^27^ were prepared in HN buffer (10 mM HEPES, 150 mM NaCl) as described previously^28^. Nano-complexes containing 10 µg siRNA in 150 µl (UT-SCC-42B) or 15 µg siRNA in 150 µl (UT-SCC-14 experiment) were injected into the peritoneal cavity 3×/week over a period of 3 weeks. Tumor size was routinely measured in all three dimensions. Upon termination of the experiment, mice were sacrificed and tumors were immediately explanted. Pieces of the tumor tissues were directly snap-frozen for preparing protein lysates or fixed in 4% paraformaldehyde for immunohistochemistry.

### Statistics

All experiments were run in biological and experimental replicates. Total n numbers (from biological and experimental replicates) per cell line were as follows: cell count, 4 – 8; colony assays, 4 – 12; spheroid assays, 10 – 20; apoptosis assays, 9; cell cycle rates, 3 – 5; molecular effects on transcript levels, 4 – 8; caspase 3/7 inhibitor assay, 9; radiosensitivity assay, 3; Western blot, 9; ELISA, 3. Statistical analyses were performed by one-way ANOVA / Tukey’s post hoc test or Student’s *t*-test using SigmaPlot 10 (Systat, Chicago, IL), with *= p < 0.05 and **= p < 0.01 and ***= p < 0.001.

## Results

### Expression and siRNA-mediated knockdown of SATB1 in HNSCC cell lines

**Figure 1:**
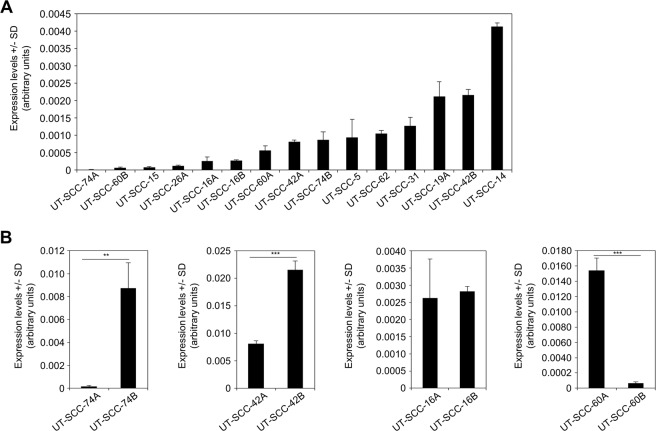
Marked differences in SATB1 (over-) expression levels between various HNSCC cell lines. (*A*) Determination of mRNA expression levels in 15 cell lines. (*B*) Matching pairs of cell lines derived from a primary tumor (left bars, indicated by ##A) and its corresponding metastasis (right bars; ##B) reveal the absence of a correlation.

Fifteen primary HNSCC cell lines of different origin (Suppl. Table 1) were monitored in 2-D culture for *SATB1* expression. In some cases, matching pairs from the primary tumor (‘A’) and from a metastasis (‘B’) of the same patient were available. Major differences were detected in *SATB1* transcript levels analyzed by RT-qPCR, with a > 300-fold range between highest (UT-SCC-14) and lowest values (UT-SCC-74A, -60B; Fig. 1A). No correlation between *SATB1* expression and original tumor location or subtype was observed (compare Fig. 1 with Suppl. Table 1). Also, there was no systematic or consistent difference in the SATB1 expression pattern of cells derived from primary tumors or metastatic sites. In some cases, cell lines originated from metastases showed considerably higher SATB1 expression than the corresponding cell line from the primary cancer (UT-SCC-74A/B, UT-SCC-42A/B) whereas no difference (UT-SCC-16A/B) or the opposite (UT-SCC-60A/B) was seen in other cases (Fig. 1B).

For subsequent studies, seven cell lines were chosen based on their *SATB1* level and origin, (i) to cover the entire spectrum from very high to very low expressing cells and (ii) to include cells derived from primary tumors and from metastases. Western blot analyses confirmed that the SATB1 protein levels in principle reflect the mRNA expression pattern, with UT-SCC-14 and -42B showing highest values, UT-SCC-60A and -5 as intermediates, and UT-SCC-16B with low expression (Suppl. Fig. 1B). Protein levels were somewhat higher than expected only in the UT-SCC-15 and -60B cells. Western blots also revealed SATB1 levels were slightly higher in xenograft tumors derived from UT-SCC-14 cells (see below) as compared to normal mucosa from tonsillitis patients (Suppl. Fig. 1C). Transcription levels of SATB2 were measured in the cell lines as well (Suppl. Fig. 1A) and did not correlate with SATB1 (Suppl. Fig. 1D). This was also true for SATB2 protein levels (Suppl. Fig. 1E which correlated well with *SATB2* mRNA (Suppl. Fig. 1A) but substantially differed from SATB1 protein levels (Suppl. Fig. 1B). This indicates that SATB2 is unlikely to be functionally linked to SATB1 in HNSCC cells.

For functional analyses, siRNA-mediated gene knockdown was performed using an siRNA identified earlier from a larger set of different siRNAs to be specific and potent^29,30^. More specifically, different SATB1-specific siRNAs had been tested previously (see Suppl. Figure 2A from^29,30^ for the two most potent examples), and ‘si467’ was selected for further experiments. Independent of endogenous expression levels and comparable to previous results in colon carcinoma cells, profound SATB1 knockdown was achieved in all cell lines as verified on the mRNA level, with a ~80–90% knockdown efficacy (Suppl. Fig. 2B). The comparison of wildtype (untreated) with negative control transfected cells (siCtrl) revealed little or no non-specific transfection effects on *SATB1* levels. The decrease in target gene mRNA upon knockdown clearly translated into a profound reduction in SATB1 protein levels, as detected in Western blot (Suppl. Fig. 2C). Notably, this was also true for longer time points (120 h; Suppl. Fig. 3B). Likewise, immunocytochemistry upon siSATB1 transfection revealed decreased SATB1 immunoreactivity, with the expected nuclear staining pattern (Suppl. Fig. 2D), and already indicated anti-proliferative effects which were subsequently analyzed in more detail.

**Figure 2:**
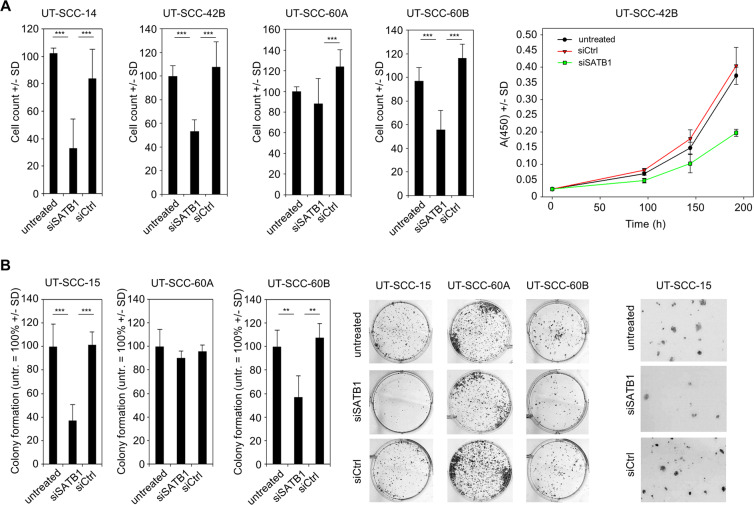
Tumor cell-inhibitory effects upon SATB1 knockdown are dependent on the cell line, but not on initial SATB1 expression levels. Cells after transient siRNA-mediated knockdown (siSATB1) are compared to negative control transfected (siCtrl) or untransfected cells. (*A*) Anchorage-dependent proliferation (endpoints after 72-96 h or growth curves), (*B*) colony formation on plastic (left: quantitation; center: original pictures of the corresponding wells; right: higher magnification).

### Effects of SATB1 knockdown on cell viability and culture growth

The transient siRNA-mediated knockdown of SATB1 led to a > 60% reduction in anchorage-dependent proliferation of UT-SCC-14 cells, as determined after 72-96 h (Fig. 2A, left). Notably, however, tumor cell inhibition was strongly dependent on the cell line. While similarly strong proliferation-inhibitory effects were observed in UT-SCC-15, -42B and -60B cells, siRNA-mediated SATB1 knockdown in UT-SCC-16B and -60A cells resulted in a less profound decrease in viable cells (Fig. 2A, Suppl. Fig. 3A). Inhibition of cell proliferation was observed over a longer time period, as exemplified by the growth curves of UT-SCC-42B cells (Fig. 2A, right). This is also supported by the analysis of SATB1 expression levels after siRNA transfection of cells, revealing an early onset (after 24 – 48 h) and long-lasting SATB1 knockdown (for at least 120 h; see Suppl. Fig. 3B). Hence it can also be concluded that the exact time point of analysis of SATB1 knockdown effects is less critical. Interestingly, major differences were also found between ‘matching pairs’: while the parent cell line UT-SCC-60A was not affected by SATB1 knockdown, a ~ 50% inhibition was observed in its counterpart (UT-SCC-60B) derived from the metastasis (Fig. 2A). Notably, these differences cannot be explained by divergent initial SATB1 expression levels, since profound anti-proliferative effects were observed in SATB1 high expressing UT-SCC-14 cells and in UT-SCC-15 or -60B cells with lower SATB1 levels (compare to Fig. 1A and Suppl. Fig. 1B).

**Figure 3:**
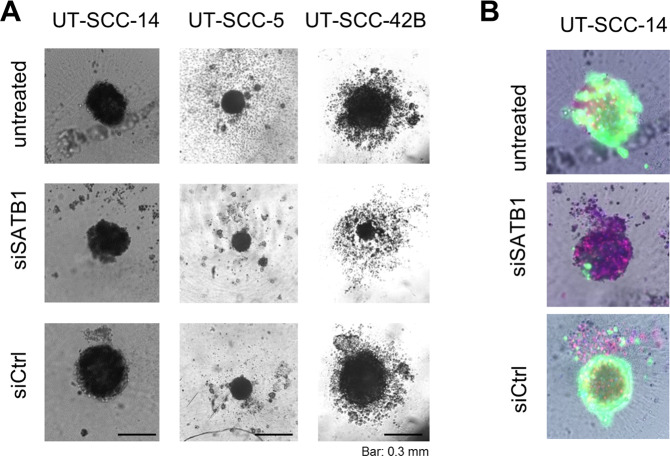
Inhibitory effects of SATB1 knockdown on tumor cell spheroids. (*A*) Spheroid formation, growth and shape, (*B*) staining of spheroids with calcein (green) for viable cells and propidium iodide (red) for dead cells.

These findings were confirmed in colony formation assays. Again, profound inhibitory effects upon SATB1 knockdown were observed in UT-SCC-15 and in UT-SCC-60B cells, but not in its parent cell line UT-SCC-60A (Fig. 2B). The quantitation did not cover very small colonies (which were mainly observed in the untreated and the negative control transfected cells) and thus tended to rather under-estimate the effects seen on the plates (compare bar diagrams with original pictures and esp. the higher magnification in Fig. 2B, right). Likewise, the decrease in colony forming capacity was rather intermediate or essentially absent in UT-SCC-42B and -16B cells, respectively, whereas SATB1 knockdown in UT-SCC-5 cells revealed a more prominent ~50% reduction of colony formation (Suppl. Fig. 3C). Interestingly, different effects were observed in spheroid assays. Only UT-SCC-42B, UT-SCC-14, UT-SCC-5 and UT-SCC-16B cell were able to form spheroids with our protocol. While the largely absence of spheroid inhibition upon siRNA transfection in UT-SCC-5 cells could be attributed to the overall rather poor spheroid growth, some effects were seen in UT-SCC-14 but not in UT-SCC-16B spheroids (Fig. 3A, Suppl. Fig. 3D). However, these were only moderate despite their strongly decreased proliferation (see above). Quite in contrast, SATB1 knockdown resulted in a very profound inhibition of UT-SCC-42B spheroid growth, leading to much smaller and less dense spheroids (Fig. 3A, right). Notably, even seemingly similar spheroids were in fact impaired by SATB1 knockdown: staining of UT-SCC-14 spheroids with propidium iodide and calcein to discriminate viable and dead cells revealed that spheroids of apparently similar size and shape consisted almost exclusively of dead cells in the siSATB1 wells (<5% cell viability) as opposed to the highly viable control spheroids (WT, siCtrl) (Fig. 3B).

### Induction of cell cycle alterations and apoptosis

**Figure 4:**
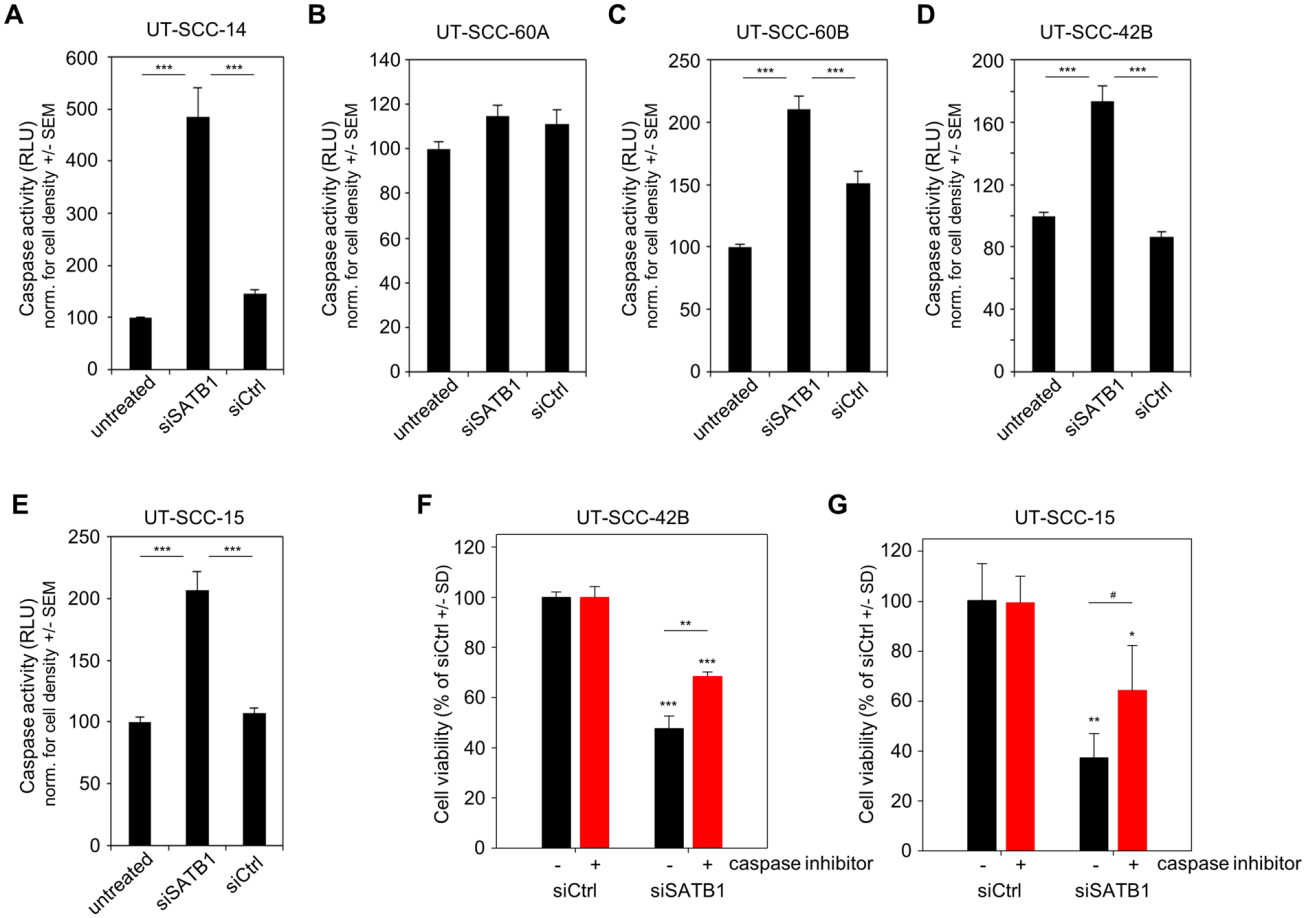
Induction of apoptosis upon SATB1 knockdown is dependent on the cell line. (*A*) – (*E*) Caspase-3/-7 activities as molecular readout downstream of both extrinsic and intrinsic apoptotic pathways in four different cell lines. (*F*) – (*G*) Direct comparison of reduced cell viabilities upon SATB1 knockdown in the presence vs. absence of caspase-3 inhibitor.

Induction of apoptosis was confirmed in cells grown on plastic via caspase-Glo^®^ assay, measuring the activation of the effector caspases-3/-7 downstream of both extrinsic and intrinsic apoptotic pathways. SATB1 knockdown led to a profound>4-fold increase in caspase-3/-7 activity in UT-SCC-14 cells (Fig. 4A), which complements very well the growth inhibitory effects. Concomitantly, induction of caspase-3/-7 was also detected in UT-SCC-60B and UT-SCC-42B but not in UT-SCC-60A cells (Fig. 4B–D), again in line with the presence/absence of growth inhibition of 2-D cultures upon SATB1 knockdown. Notably, even the distinct growth response of the parent primary tumor-derived and the metastasis-derived cell lines UT-SCC-60A and -60B, respectively, was reflected in the caspase activation. Again, differences cannot be explained by different endogenous expression levels: for example, a profound 2-fold increase in active caspase-3/-7 was obtained in UT-SCC-15 cells upon siSATB1 transfection (Fig. 4E), but not in UT-SCC-16B cells (1), despite very similar (low) SATB1 expression levels. A strong induction of apoptosis upon SATB1 knockdown was also observed in UT-SCC-5 cells with intermediate SATB1 expression (Suppl. Fig. 4B).

**Figure 5:**
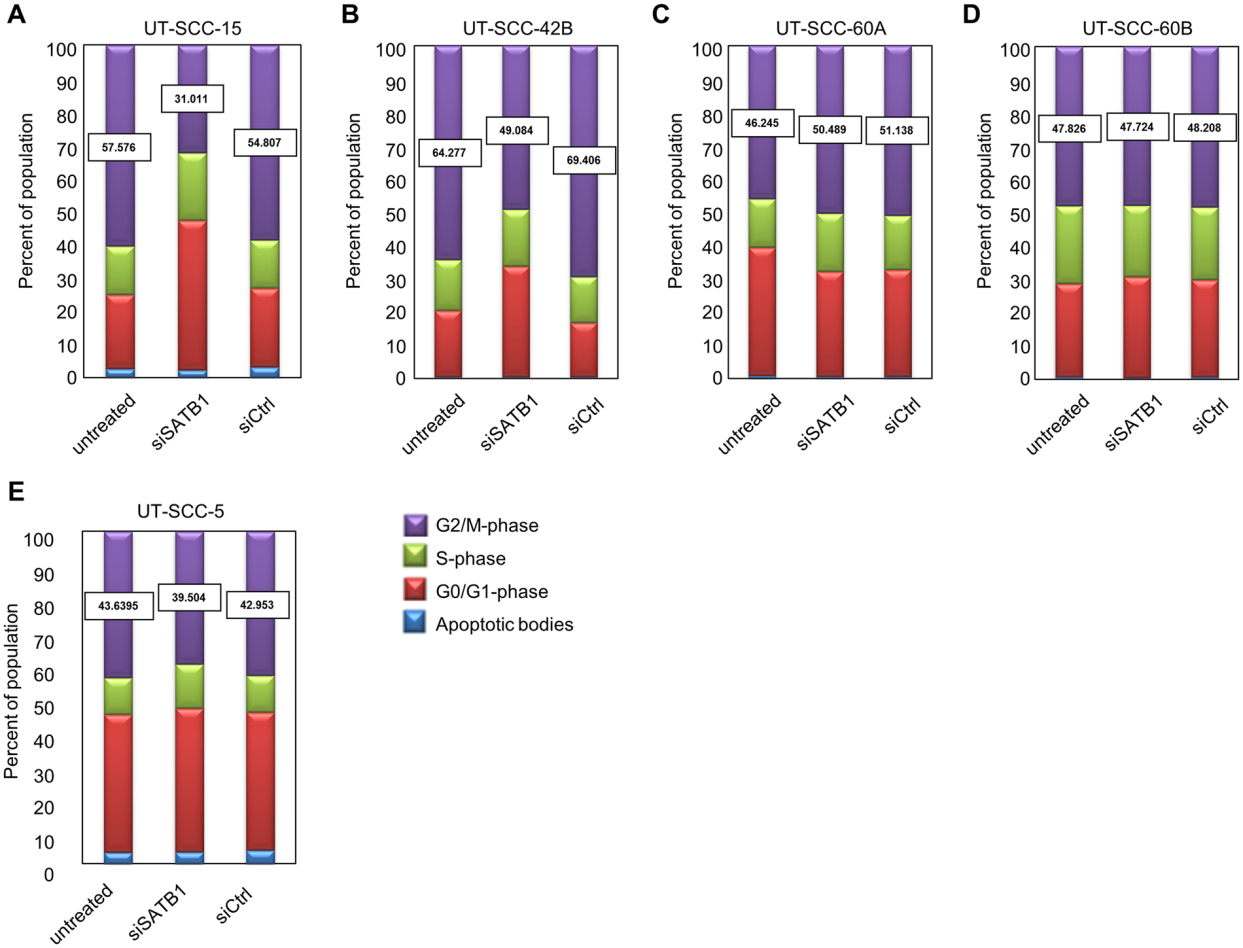
SATB1 knockdown mediates alterations in cell cycle dependent on the cell line. (*A*) – (*E*) Cell cycle distribution in five different cell lines transfected as indicated on the x-axis 72 h prior to addition of nonocodazole for 12-18 hours for inducing G2/M arrest. Higher G2/M percentages thus indicate a more rapid cell cycle progression, with more cells reaching the nocodazole-induced G2/M block in the given time frame.

To further confirm the relevance of caspase activation upon SATB1 knockdown as relevant mechanism for the decrease in cell viability, additional experiments in the presence vs. absence of an apoptosis inhibitor were performed. The direct comparison of siSATB1-mediated reduction of cell viability + /− caspase-3/-7 inhibitor revealed that the addition of the caspase inhibitor partially antagonized the decrease in cell viability upon siSATB1 transfection. This was found in UT-SCC-42 as well in UT-SCC-15 cells (Fig. 4F,G). These findings support the functional role of caspase-3/-7 activation upon SATB1 knockdown, while not being the only effect. In fact, SATB1 knockdown also led to a rather profound cell cycle deceleration, as determined by flow cytometry. After siRNA transfection and upon the onset of the specific knockdown (72 hours), nocodazole was added for inducing a G2/M arrest. 12-18 hours later the cell cycle distributions were determined. Thus, the higher the proportion of cells in G2/M phase in Fig. 5A, the more rapid was cell cycle progression, with more cells reaching the nocodazole-induced G2/M block in the given time frame. siSATB1 transfection in UT-SCC-15 and UT-SCC-42B cells lowered the proportion of cells reaching the G2/M block indicating slower cell cycle progression (Fig. 5A, center bars). No difference was observed in UT-SCC-60A cells, which is again in line with the absence of growth inhibition in this cell line. However, the same is true for the UT-SCC-60B cells in spite of growth inhibitory effects. Similarly, cell cycle deceleration only contributed to a minor extent in UT-SCC-5 cells while induction of apoptosis was identified as major driver of siSATB1-related growth retardation (Fig. 5E vs. Suppl. Fig. 4B). Taken together, our data indicate that the growth inhibition upon SATB1 knockdown seen in some HNSCC cell line models results from both, enhanced apoptosis and cell cycle deceleration, whereas in others induction of apoptosis turned out as the main underlying mechanism.

To evaluate if these cellular effects also translate into an altered sensitivity to irradiation, UT-SCC-42B cells which had shown both, induction of apoptosis and deceleration of cell cycle upon SATB1 knockdown were applied in a radioresponse assay. Upon efficient SATB1 knockdown (Suppl. Fig. 5A), a ~50% reduction in colony formation was observed (Suppl. Fig. 5B). Exposure of the cells to single dose X-ray led to a characteristic dose-dependent decrease in clonogenic cell survival. However, cellular radiosensitivity was independent of SATB1 expression levels as SATB1 knockdown did not modify the dose-effect curves (Suppl. Fig. 5C). The same was seen in another HNSCC cell line (FaDu) not included in the present study.

### Molecular effects upon SATB1 knockdown

**Figure 6:**
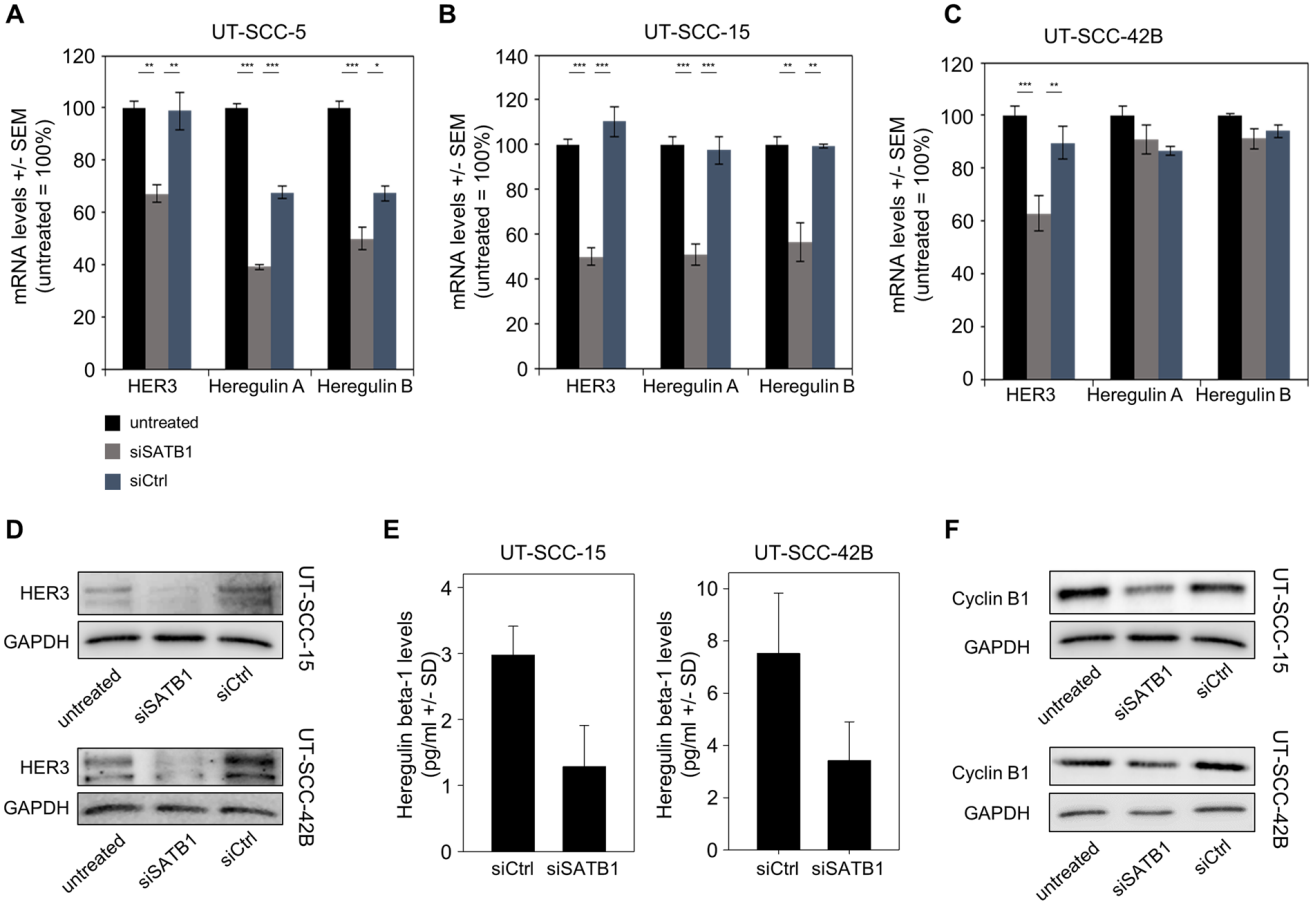
SATB1 knockdown-mediated alterations in expression levels of various genes. (*A*) – (*C*) mRNA levels of HER3 and its ligands Heregulin A and B, as determined in three different cell lines. (D) Western blots demonstrating reduced HER3 protein levels. (*E*) Decreased Heregulin beta-1 levels, as determined by ELISA. (*F*) Western blots demonstrating reduced Cyclin B1 protein levels.

Based on previous studies on the role of HER3 in HNSCC^31^ and the increase of HER3 upon SATB1 overexpression in breast and colon carcinoma^15,29^, we analyzed *HER3* mRNA levels by RT-qPCR (Fig. 6A-C, Suppl. Figure 6A–D). Strikingly, a downregulation of *HER3* mRNA upon SATB1 knockdown was recorded in all cell lines. However, the effect was heterogeneous and the extent of *HER3* mRNA reduction in SATB1 depleted cells was cell line dependent, again with minor changes in UT-SCC-16B cells that in general had remained rather unaffected by SATB1 knockdown, while *HER3* mRNA levels were found reduced up to ~ 50% for example in UT-SCC-15 cells where siSATB1 had shown profound tumor cell inhibition. These results were also confirmed on the protein level, where Western blots from UT-SCC-15 and UT-SCC-42B cells showed profoundly weaker bands upon siSATB1 transfection (Fig. 6D). On the functional side, the observation of tumor cell-inhibition upon transfection with a HER3-specific siRNA (Suppl. Fig. 6E,F) also revealed that this effect of SATB1 knockdown on HER3 levels likely contributes to the anti-oncogenic phenotype of SATB1 inhibition.

Notably, decreased HER3 levels were often paralleled by reduced mRNA levels of the HER ligands Heregulin A and Heregulin B, except for UT-SCC-42B cells where mRNA levels remained unaltered despite HER3 downregulation (Fig. 6A-C, Suppl. Fig. 6A–D). On the protein level, however, a marked>50% reduction of Heregulin beta-1 was seen by ELISA also in this cell line, which compared well with similar results from UT-SCC-15 cells (Fig. 6E).

To further link cellular effects of siSATB1 transfection to other molecular alterations, the expression of genes related to cell cycle, epithelial-mesenchymal transition (EMT), metastasis and induction of apoptosis were determined by RT-qPCR. Cell lines were selected that had shown rather profound (UT-SCC-42B) or essentially no (UT-SCC-16B) growth inhibition upon SATB1 knockdown. Strong siRNA-mediated SATB1 downregulation was achieved in both cell lines, serving as positive control readout. In UT-SCC-42B cells, reduction in SATB1 expression was associated with a significant increase in Cyclin B1 and Cyclin D1 transcript levels (Suppl. Fig. 7A), which was somewhat counter-intuitive in the light of the observed deceleration of cell cycle under this condition. No alterations in these genes were observed in UT-SCC-16B cells (Suppl. Fig. 7B). Analysis on the protein level, however, revealed decreased levels of Cyclin B1 upon SATB1 knockdown in UT-SCC-42B cells. This was also confirmed in UT-SCC-15 cells (Fig. 6F). In UT-SCC-42B cells, mRNA levels of the anti-apoptotic survival kinase Pim1 were upregulated upon siSATB1 transfection, as well as E-cadherin and N-cadherin indicating effects of SATB1 knockdown on EMT (Suppl. Fig. 7). The downregulation of beta-catenin, a multifunctional protein that plays an important role in intercellular adhesion, may contribute to the more loosely looking shape of UT-SCC-42B spheroids upon SATB1 knockdown, and was paralleled by an upregulation of MMP-7.

Taken together, this indicates molecular alterations in UT-SCC-42B, UT-SCC-15 and other cell lines that showed cellular effects upon SATB1 knockdown, while in UT-SCC-16B cells molecular levels remained largely unchanged, reflecting the poor cellular response of this model to SATB1 downregulation.

### Therapeutic inhibition of tumor xenograft growth upon treatment with nanoparticle-formulated siRNAs

**Figure 7:**
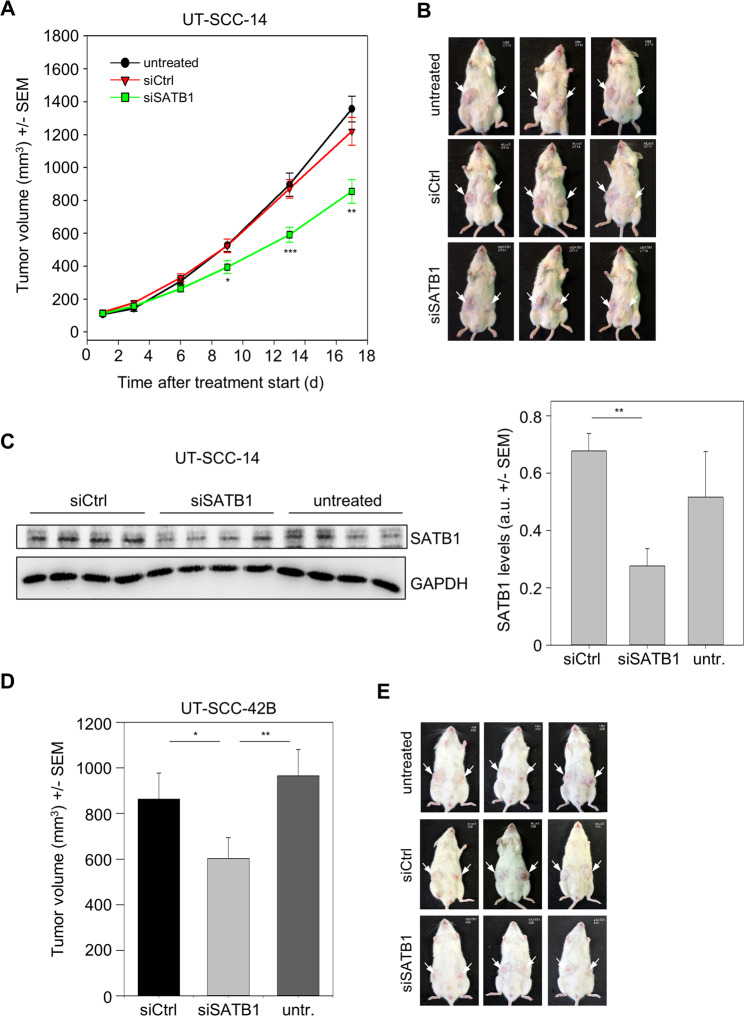
Inhibition of tumor growth *in vivo* upon therapeutic SATB1 knockdown. Subcutaneous tumor xenografts based on two cell lines were established in immunodeficient mice. Upon randomization, mice were treated by i.p. injection of 10 µg siRNAs specific for SATB1 (si467) vs. negative control siRNAs (siCtrl) or untreated. For siRNA delivery, siRNAs were formulated in polymeric nanoparticles based on a low-molecular weight polyethylenimine (PEI F25-LMW). (*A*) Growth curves of UT-SCC-14 tumors, (*B*) photos of three representative mice per group upon termination of the experiment, (*C*) quantitation SATB1 protein levels in tumor xenografts after explantation; left: Western blot showing four representative samples per group, right: quantitation of Western blot bands. (*D*) Tumor sizes and (*E*) photos of three representative mice per group at the end of the UT-SCC-42B experiment.

**Figure 8:**
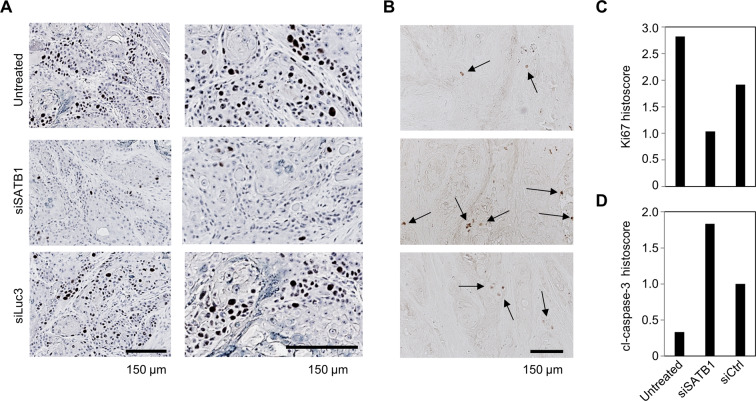
Immunohistochemical analysis of UT-SCC-14 tumor xenografts from the PEI/siRNA therapy study. (*A*) Ki-67 staining for proliferating cells (brown, with hematoxylin counterstain), (*B*) cleaved caspase-3 staining for apoptotic cells (brown, without counterstain). (*C*) Histoscores derived from the blinded rating of the signal densities (Ki-67) or (*D*) the total number of positive cells (cleaved caspase-3) in given percentages of the whole section, plotted as sum.

Beyond effects on tumor cells *in vitro*, therapy studies in xenograft mouse models were designed to test tumor-inhibitory effects of siRNA-mediated knockdown of SATB1 more rigorously *in vivo*. UT-SCC-42B and UT-SCC-14 cells were able to form s.c. xenograft tumors in immunodeficient mice. For siRNA delivery *in vivo*, polymeric nanoparticles based on polyethylenimine (PEI) were used, as established previously^30,32^. Upon establishment of tumor xenografts, mice were systemically injected 3×/week with the ~250 nm nanoscale polyplexes (10 µg siRNA/injection), and tumor xenografts were regularly measured. Over a time period of about 2.5 weeks, a ~ 14-fold increase in tumor volume was recorded both negative control groups (untreated and PEI/siCtrl). In contrast, treatment with PEI-complexed siSATB1 led to a ~40% inhibition of tumor growth (Fig. 7A; see Fig. 7B for representative mice). Differences in tumor volumes were confirmed in the explanted tumors upon termination of the experiment (not shown), which were also used for analysis of target gene expression. Western blots revealed a ~50% knockdown of SATB1 in the specific treatment group as compared to the negative controls (Fig. 7C). In another experimental series with UT-SCC-42B cells, similar tumor-inhibitory effects upon systemic PEI/siSATB1 treatment of mice were observed (Fig. 7D,E). The explanted UT-SCC-14 tumors were also analyzed for (changes in) proliferation and apoptosis upon SATB1 knockdown. Immunohistochemistry (IHC) for the proliferation marker Ki67 revealed a marked reduction of the number of proliferating cells in the PEI/siSATB1 treatment group as compared to tumors from untreated or negative control treated mice (Fig. 8A). The quantitative assessment of the Ki67 histoscore indicated an at least 50% decrease in cell proliferation (Fig. 8C). Likewise, the number of apoptotic cells as identified by IHC using an antibody against cleaved caspase-3 was found increased in the specific treatment group (Fig. 8B,D). Thus, it can be concluded that the observed tumor-inhibitory effects rely on both, anti-proliferative and pro-apoptotic effects of SATB1 knockdown.

## Discussion

While previous studies have already suggested SATB1 as putative prognostic marker in HNSCC^19–21^, the analysis of its functional role so far rested on a very few cell lines, thus not addressing possible intra- and inter-tumor heterogeneity. Indeed, when analyzing knockdown effects in various primary cell lines, different effects upon SATB1 knockdown were observed. Likewise, major differences in expression levels were found, even in matching pairs (primary tumor/metastasis). Notably, while in some cases SATB1 expression was higher in cells derived from metastases compared to their primary tumor counterparts, as expected from the literature^20^ but not from TCGA data (see Introduction), the opposite relation was found in other pairs. Thus, despite the fact that SATB1 overexpression has been associated with increased metastasis, this may not necessarily translate into higher expression levels as compared to the parental tumors. The somewhat contradictory findings published in the literature and in databases regarding SATB1 overexpression in solid tumors may also indicate that expression levels might not be the most important parameter for defining its (tumor-)biological relevance. Indeed, while our functional assays revealed that cell culture only 7 out of 15 cell lines show reduced growth upon SATB1 knockdown, with rather mild inhibitory effects in some models, mRNA or protein expression levels of SATB1 did not determine the magnitude of anti-proliferative effects. Concomitantly, SATB1 levels in UT-SCC-14 tumor xenografts were found to be only slightly elevated over normal mucosa from tonsillitis patients. Taken together, this indicates that the mere determination of SATB1 expression levels may not be a sufficient predictor of oncogenic relevance, as also seen in other cases (e.g., the oncogenic survival kinase Pim1^33^). In particular, it must be concluded that somewhat lower expression levels may not necessarily exclude SATB1 from being an attractive target for inhibition in HNSCC therapy. On the other hand, it remains to be seen if and to what extent SATB1 inhibition will achieve therapeutic efficacies in ‘real’ tumors, beyond tumor cell lines and their corresponding xenograft tumors. The compilation of our data suggests that this may also depend on the more general molecular setup of the target cells, e.g., with regard to expression levels of oncogenes like HER3. In fact, this might explain different sensitivities of tumors/ tumor cells towards SATB1 inhibition. Based on this, one can also conclude that SATB1 inhibition in normal cells may not necessarily lead to (unwanted) effects in these cells similar to the desired effects in tumor cells, since this may well rely on the levels of genes affected by SATB1 and its inhibition. This is for example true for HER3 which we identified to be regulated by SATB1 and to be functionally relevant in HNSCC cells, but which is barely expressed in normal tissue, independently from SATB1.

Also, our results emphasize that tumor cell-inhibitory effects may not be readily seen in 2D culture on plastic, representing a rather artificial system, but require 3D systems (e.g., spheroid assays) that resemble more closely the *in vivo* situation. Still, however, major differences were seen between different cell lines, indicating that more subtle differences in the molecular or cellular context of the tumor cell may determine to what extent SATB1 expression is pivotal in HNSCC tumorigenesis.

Therapeutic effects in terms of decreased numbers of viable cells upon SATB1 inhibition/knockdown may be based on the induction of apoptosis or deceleration of cell cycle, or both. Notably, all scenarios were found in our experiments, again dependent on the HNSCC cell line model. This is in line with the notion of SATB1 affecting the expression of many genes, as to be readily expected from its broader molecular mechanism(s) of action as chromatin organizer and as shown previously in other tumor entities. SATB1 has been shown previously in other tumor entities to influence the expression of HER receptor family members which were sometimes, but not always, found affected by SATB1 overexpression or inhibition (^15,29,30^; see^17^ for review). Targeting the HER receptor family is already in practice for treatment of HNSCCs^34^. Among the four members of this family, HER3 is the only membrane receptor that does not possess a tyrosine kinase cytoplasmic domain and thus requires heterodimerization with HER1 or HER2^35,36^, with these heterodimers being particularly active. Indeed, studies on HNSCC have been published showing increased chemosensitization^37^, radiosensitization^38^ and susceptibility to HER1 inhibition (Cetuximab)^36,38,39^ after inhibiting the HER3 receptor. Thus, while various HER receptors are found overexpressed or aberrantly activated by mutations, and are responsible for mitogenic signaling, the HER3 receptor is of particular interest. It is overexpressed not only in HPV-negative^40^ but also in HPV-positive tumors^41^ which further increases its relevance when it comes to the treatment of HNSCCs. Thus, the downregulation of HER3 upon SATB1 knockdown may be particularly relevant. This is even more so since we found SATB1 to influence in parallel the expression of the HER3 ligands, Heregulin alpha and beta. Consequently, SATB1 knockdown inhibits the autocrine or paracrine HER3 activation on two levels (receptor + ligand). SATB1 (inhibition) is expected to affect several target genes of interest. Beyond coding genes, SATB1 may also interfere with the expression of non-coding genes, thus leading to the aberrant expression of oncogenic miRNAs or lncRNAs. Moreover, the fact that HER ligands are not only expressed in tumor cells, but also in adjacent tumor stroma cells, indicates that effects obtained in ‘pure’ tumor cell models (even 3-D) may rather underestimate therapeutic efficacies of SATB1 inhibition. Still, our results as well as previous studies indicate that the sole targeting of SATB1 may not be sufficient for therapeutic intervention, thus requiring combination with radiation or chemotherapy. While in our experiments no radiosensitization was found, which is in line with previously published rather mild effects on chemosensitivity towards docetaxel upon SATB1 knockdown^24^, this may again depend on the assay, the cell model and cellular context provided, and the treatment regime chosen in the present study. At least, no adverse effect of SATB1 downregulation on radioresponse was seen which could in fact result from a pronounced cell cycle arrest, in particular in a more radioresistant cell cycle phase. It has to be kept in mind, that actively proliferating cells are in general more susceptible to DNA-damaging factors than arrested cells, but then cells in G2/M phase show highest sensitivity whereas G1-phase cells are more resistant^42^. In the context of standard-of-care for HNSCC patients with the majority receiving radiochemotherapy in the course of their disease, the actual contribution of SATB1 inhibition to radio- and/or chemosensitivity, or to avoiding secondary resistance during therapy, remains to be elucidated in greater detail in *ex vivo* and *in vivo* models.

In the absence of small molecule inhibitors of SATB1, which may not be easy to generate based on the structure of the protein and its mechanism of action, we employed RNAi-mediated gene knockdown. The use of our polymeric nanoparticles developed previously for siRNA or miRNA delivery (^28,43^; see^44^ for review) allowed us to also study SATB1 knockdown effects *in vivo*, and to confirm *in vitro* data with regard to tumor inhibitory effects, decrease in tumor cell proliferation and induction of apoptosis. Their formulation in a nanoparticle system addresses several shortcomings of small RNAs as drugs, i.e., poor stability, cell uptake and tissue penetration as well rapid renal clearance and excretion. Notably, most recently the first siRNA drug has obtained market approval^45^, thus clearly indicating the possibility of exploring siRNAs for RNAi-mediated oncogene knockdown. It must be kept in mind, however, that SATB1 also exerts physiological functions in different organs, for example maintaining pluripotency and preventing exhaustion of the hematopoietic stem cells. Thus, even when avoiding off-target effects on other genes by careful selection of an appropriate siRNA, possible side effects upon systemic application will have to be analyzed in detail. The transient nature of siRNA therapies, however, may be well suited for handling those while exploring the desired effects of SATB1 knockdown in tumor cells, benefitting from its actions on several molecular targets in parallel.

## Supplementary information

Supplementary Information.

## Data Availability

All data is available in the main paper and its supplementary information
