# Supplementary material for: SATB1 as oncogenic driver and potential therapeutic target in head & neck squamous cell carcinoma (HNSCC)
Source: Sci Rep. 2020 May 25;10:8615. doi: 10.1038/s41598-020-65077-y (PMC7248088; doi:10.1038/s41598-020-65077-y)
Supplement: Supplementary file 1 — Supplementary Information. [file 41598_2020_65077_MOESM1_ESM.pdf]

SATB1 as oncogenic driver and potential therapeutic target in head & neck squamous cell carcinoma (HNSCC)

Omkar Panchal[1], Gunnar Wichmann[2], Reidar Grenman[3], Lisa Eckhardt[4], Leoni A. Kunz-Schughart[4,5], Heike Franke[6], Andreas Dietz[2] & Achim Aigner[1,7]

Supplementary Information

Suppl. Materials and Methods

Radiosensitivity assay

In the radiosensitivity assays conditions were 8% CO_2 and humidified atmosphere. Media / supplements were from Biotech, Aidenbach, Germany. Cells were cultured only for two months which accounted for 12-16 passages, after which a new culture was thawed from the stock.

Radioresponse was assessed in clonogenic survival assays. Single cell suspensions prepared from exponentially growing monolayer cultures pre-exposed for 72h to siLuc3 or siSATB1 in transfection reagent as described above were seeded into 6-well plates at defined densities (300 – 4,800 cells/well) in supplemented standard DMEM. After a 10 hours incubation interval to allow cell adherence, culture plates were irradiated at room temperature using single doses of 0-10 Gy with a dose-rate of about 1.3 Gy/min (200 kV X-rays; Yxlon Y.TU 320, Yxlon, 0.5 mm Cu filter). UT-SCC-42B and FaDu cells were then cultured for a total of 8 and 10 days, respectively, to guarantee >5 doublings and the formation of colonies consisting of ≥50 cells. Colonies were fixed and stained followed by manual microscopic counting to determine plating efficiencies (PEs). Survival fractions (SFs) relative to control conditions at 0 Gy were calculated and clonogenic survival curves were fitted, employing the linear-quadratic model $SF = e^{-(A \times D + B \times D^2)}$ (D: irradiation dose; A and B: variables defining the irradiation dose) as described in [1].

Clonogenic surviving fractions were statistically compared by independent Mann-Whitney-*U* tests including Bonferroni-Holm correction for multiple testing, while linear regression was used for statistical evaluation of the parameters A and B in the linear-quadratic functions modeling the irradiation dose dependent clonogenic survival curves. These tests were performed using SPSS Statistics 21 (IBM Corporation, NY) [1].

Western Blotting

For protein isolation, identical amounts of cells in suspension (500,000 cells, harvested by trypsinization and counted in a Neubauer chamber) were lysed or, if cell numbers in the wells were substantially different, the protein concentration of the total cell lysates (generated from cells in monolayer) was measured using the BioRad DC protein quantification kit (BioRad Laboratories, Munich, Germany). RIPA-SDS buffer (50 mM Tris HCl pH 7.4, 150 mM NaCl, 1% (v/v) TritonX-100, 0.5% (w/v) sodium deoxycholate, 0.1% (w/v) SDS, 1 mM EDTA, 10 mM NaF) was used for lysing the cells. Tumor homogenization was done with a tissue homogenizer (Ultra-turrax; IKA, Staufen, Germany). Cells in monolayer were scraped by cell scraper (Sarstedt; Nümbrecht, Germany) and cells in suspension were lysed only by pipetting. After homogenization (tumor/cell suspension/scrapped cell lysate), undissolved cell debris was eliminated by centrifugation at 5,000 rpm for 10 min at 4 °C. For sodium dodecyl sulfate polyacrylamide gel electrophoresis (SDS-PAGE), 30 µg total protein was separated by SDS-PAGE, prior to transfer onto a 0.45 µm PROTRAN®

nitrocellulose membrane (Whatman, Dassel, Germany). Membranes were blocked with 5% (w/v) non-fat dry milk in TBST (10 mM Tris/HCl, pH 7.6, 150 mM NaCl, 0.1% Tween 20), washed with TBST and incubated overnight with primary antibodies at 4°C. For details on the specific antibodies used for immunodetection, see Suppl. Table 3. GAPDH or actin were used for loading control. After washing with TBST, membranes were incubated with horseradish peroxidase-coupled secondary antibodies conjugated with HRP for detection. Protein bands were visualized by using the chemiluminescence ECL kit from Thermo Fisher Scientific in a LAS-4000 chemiluminescence detection system (GE Healthcare, Munich, Germany). Immunoblots of SATB2 and its GAPDH loading controls were visualized using a secondary antibody conjugated with a NIR fluorophore. The detection was done in the near infrared region (700 and 800 nm) using an Odyssey Fc apparatus (LI-COR, Lincoln, NE). Densitometric analysis of Western blots was performed using ImageJ software, and expression levels were normalized to GAPDH.

Immunofluorescence microscopy

5,000 cells in 500 µl of complete medium were seeded per compartment on Superfrost excel slides (Thermo Fisher Scientific). Compartments for several independent samples were performed by using flexiPERM® reusable silicone inserts for compartmentalizing the growth surface (Sarstedt, Nümbrecht, Germany). 24 h after seeding, cells were transfected with 5 nM siRNA in 600 µl media as described above. Cells were fixed 96 h later with 100% ice cold methanol for 30 min at -20 °C. Antigen retrieval was done by boiling the slides for 10 min in 1x citrate buffer, pH 6.0 (Dako/Agilent, Santa Clara, CA), prior to washing in PBS. Unspecific binding was blocked by exposure to 5% (v/v) goat serum, 1% (w/v) BSA in PBS followed by incubating the slides with antibodies against SATB1 (EPR 3951, Abcam) at 4 °C overnight. In parallel, slides without primary antibody were run as negative control. After washing with PBS 3 times for 5 min, Cy3-labelled anti-rabbit antibodies (Jackson Immunoresearch, Ely, UK) were added and incubated at room temperature for 1 h, again followed by washing. To stain cell nuclei, the slides were incubated with 40 µg/ml Hoechst 33342 in TBS (Molecular Probes, Leiden, The Netherlands) for 10 min at room temperature. Images were taken with a confocal microscope (Leica SP8 confocal microsope; Leica, Wetzlar, Germany).

Immunohistochemistry

Tissues were fixed in 4% paraformaldehyde, prior to dehydration and paraffin embedding. Paraffin sections were fixed on the slide by baking at 56 °C overnight and microwaving for 5 min. For de-paraffination, slides were incubated 2 x 5 min in Neo-Clear (Merck, Darmstadt, Germany), followed by a series of decreasing ethanol concentrations (100%, 96%, 90%, 70%) and a final short washing step in distilled water. Antigen retrieval was done by microwave pre-treatment in 10mM sodium citrate buffer, pH 6.0, at 90 °C for 15 min. After cooling at room temperature for 10 min, slides were washed 2 x 5 min in PBST (PBS + 1% Tween-20). After washing 2 x 5 min in PBST, slides were

treated for 15 min at 4°C with 0.3% H_2O_2 in PBST, to block endogenous peroxidase activity. For blocking of nonspecific binding sites, slides were treated with blocking solution (PBST containing 2% bovine serum albumin, 10% normal serum from the secondary antibody source) at room temperature for 30 min, followed by incubation with the primary antibodies (mouse anti-Ki67 (1:100) or mouse anti-cleaved caspase-3 (1:50; see Suppl. Table 3 for details) in PBST/2% bovine serum albumin overnight at 4°C. After intensive washing in PBST, slides were incubated with a biotinylated anti-mouse or anti-rabbit secondary antibody (immunoglobulin IgG (H+L); 1:100; Vector Laboratories, Burlingame, CA) in PBST / 2% bovine serum albumin for 1 h at room temperature. For detection, slides were incubated with Avidin-Biotin complex solution (DAKO, Glostrup, Denmark) for 30 min at 37 °C. For visualization, slides were washed and then incubated in 1% 3,3´-diaminobenzidine tetrahydrochloride (DAB; Sigma-Aldrich) / H_2O_2 in PBST until the brown staining was observed under the microscope. Where applicable, sections were counterstained with hematoxylin, priot to dehydratíon in a series of graded ethanol, processing through n-butylacetate (Carl Roth, Karlsruhe, Germany) and coverslipping with Entellan (Merck). Control experiments were carried out without the primary antibody. Histoscores were derived from the blinded rating of the signal densities (Ki-67) or the total number of positive cells (cleaved caspase-3) in given percentages of the whole section, and are plotted as sum.

SiRNA sequences

Sequences were as follows: siSATB1 (‘467’) Sense: 5‘- GCU UCA AGA UGU GUA UCA UdTdT-3‘, Antisense: 5’- AUG AUA CAC AUC UUG AAG CdTdT-3’; siSATB1 (‘989’) Sense: 5’- GUA UGC AGU GAA UAG ACU UdTdT -3’, Antisense: 5’- AAG UCU AUU CAC UGC AUA CdTdT -3’; siLuc3 (serving as negative control); sense: 5′- CUU ACG CUG AGU ACU UCG AdTdT -3′, antisense: 5′- UCG AAG UAC UCA GCG UAA GdTdT -3′; siHER3: 5’- CCU UGA GAU UGU GCU CAC GTT -3’.

Colony formation and spheroid assay

For assessing the ability of the cells to form colonies on plastic, cells were trypsinized 72 h after transfection, counted as described above, and 1,000- 2,000 cells were seeded in a 6 well plate. Cells were allowed to grow for 5 – 8 days until each colony contained ~ 30 - 50 cells, prior to staining with 1% (w/v) methylene blue in 70% (v/v) ethanol solution for 45 to 60 min. Stained colonies were washed with distilled water, air dried, photographed and counted by image J.

For spheroid assays, transfected cells were trypsinized, counted, and 5,000 cells per well in 250 µl medium were seeded in Nexcelom 3D 96-well round bottom Ultra-low attachment plates (Nexcelom Bioscience, Lawrence, MA). Spheroids were allowed to grow for two weeks prior to taking photos. For live/dead stain, UT-SCC-14 spheroids were stained by adding 50 µl of complete media containing (total volume 300 µl) 5 µM Calcein AM (Thermo Fisher Scientific), 1 ng propidium iodide (Sigma-

Aldrich) and 6 ng Hoechst 33342 dye (Thermo Fisher Scientific). Images were recorded using a Celigo Imaging Cytometer (Nexcelom).

Apoptosis assay

Apoptosis was determined by detection of active Caspase 3/7 using a bioluminescent Caspase 3/7 Glo® assay (Promega, Mannheim, Germany). For this purpose, cells were seeded in 96 well plates at an initial density of 1,000 cells/well and transfected as described above. Detection of Caspase 3/7 activation was performed 72 h post transfection according to the manufacturer's protocol. Luminescence was measured using a POLAR star Omega reader (BMG Labtec, Jena, Germany) after 1 h of incubation at room temperature in the dark. In parallel, cell numbers were determined using a WST-1 colorimetric assay (Roche, Mannheim, Germany), and used for the normalization of caspase luminescence results.

Caspase inhibition assay

Cells were seeded in a 96 well plate at density of 1000 or 2000 cells/well in 250 µl serum deficient media (2% FCS for UT-SCC-42B and 5% FCS for UT-SCC-15). Cells were transfected with 5 nM siRNA in 260 µl media, and caspase 3/7 inhibitor (Enzo Life Sciences, Lörrach, Germany; 20 µM or 25 µM for UT-SCC-42B or UT-SCC-15 cells, respectively) was added to the wells 24 h and 48 h post transfection. The number of viable cells was monitored at 72 h or 96 h post transfection by using the CCK8 reagent (Dojindo, Munich, Germany) and measuring the absorbance at 450 nm.

Reference

1. Hinrichs CN, Ingargiola M, Kaubler T, et al. Arginine Deprivation Therapy: Putative Strategy to Eradicate Glioblastoma Cells by Radiosensitization. *Molecular cancer therapeutics* 2018;**17**:393-406.

Suppl. Figure legends and Tables

Suppl. Fig. 1. Determination of SATB1 and SATB2 expression levels in various cell lines. (A) SATB2 mRNA levels. (B) SATB1 protein levels as determined by Western blotting (bars: quantitation normalized for GAPDH, lower panels: original Western blots). (C) Comparison of SATB1 expression levels in UT-SCC14 tumor xenografts vs. normal mucosa from tonsillitis patients (the asterisk denotes a sample with lower loading amounts due to limited tissue availability). (D) Scatter plot revealing the absence of a correlation between SATB1 and SATB2 mRNA levels. (E) SATB2 protein levels.

Suppl. Fig. 2. Efficient siRNA-mediated SATB1 knockdown. (A) Comparison of different SATB1-specific siRNAs (in the colon carcinoma cell line HCT-116; taken from [28,29]). More details on the validation of efficacy and specificity of the si468 selected for subsequent experiments can be found in [28,29]. (B) Profound reduction of SATB1 mRNA upon transfection with specific (siSATB1) vs. negative control (siCtrl) siRNAs or untreated cells. The comparison between various cell lines reveals that knockdown efficacies are independent of initial SATB1 expression levels. (C) Determination of SATB1 knockdown on the protein level. Original Western blots from three different cell lines at two different time points are shown, with GAPDH used as loading control. (D) Immunocytochemistry for analyzing SATB1 levels. Beyond the decrease in SATB1 expression upon siSATB1 transfection (lower panel) vs. negative control (siCtrl, upper panel), anti-proliferative effects are observed as well (scale bar: 100 µm). Knockdown is also observed in the higher magnification; see UT-SCC-14 cells as representative example, furthermore confirming the nuclear SATB1 localization (red; compare to blue Hoechst 33342 staining of siCtrl transfected cells; scale bar: 10 µm).

Suppl. Fig. 3. Tumor cell-inhibitory effects upon SATB1 knockdown are dependent on the cell line, but not on initial SATB1 expression levels. Cells after transient siRNA-mediated knockdown (siSATB1) are compared to negative control transfected (siCtrl) or untransfected cells. (A) Anchorage-dependent proliferation, (B) prolonged reduction of SATB1 protein levels upon siSATB1 transfection, as determined by Western blot; (C) colony formation on plastic (upper panel: quantitation; lower panel: original pictures of the corresponding wells; bar: 0.3 mm), (D) spheroid formation assay.

Suppl. Fig. 4. Induction of apoptosis and effects on cell cycle upon SATB1 knockdown are dependent on the cell line. (A) – (B) Caspase-3/-7 activities in two different cell lines as molecular readout downstream of both extrinsic and intrinsic apoptotic pathways.

Suppl. Fig. 5. No alterations in cellular sensitivity towards radiation upon SATB1 knockdown. (A) Reduced SATB1 levels upon siSATB1 transfection as compared to siCtrl lead to (B) inhibition of

colony formation. (C) Cell survival upon radiation is strongly dependent on the dosage, but not affected by SATB1 knockdown.

Suppl. Fig. 6. SATB1 knockdown-mediated alterations in expression levels of HER3 and its ligands Heregulin A & B. (A) – (D) mRNA levels in four different cell lines. (E) – (F) Inhibitory effects of siRNA-mediated HER3 knockdown in two different cell lines. Lower panels: HER3 expression as determined by Western blot.

Suppl. Fig. 7. SATB1 knockdown-mediated alterations in expression levels of various genes. (A) – (B) mRNA levels of genes involved in EMT, cell cycle and cell viability.

Suppl. Table 1: Background information on cell lines used in this study

Suppl. Table 2. Sequences of primers used in this study

Suppl. Table 3: Background information on antibodies used in this study

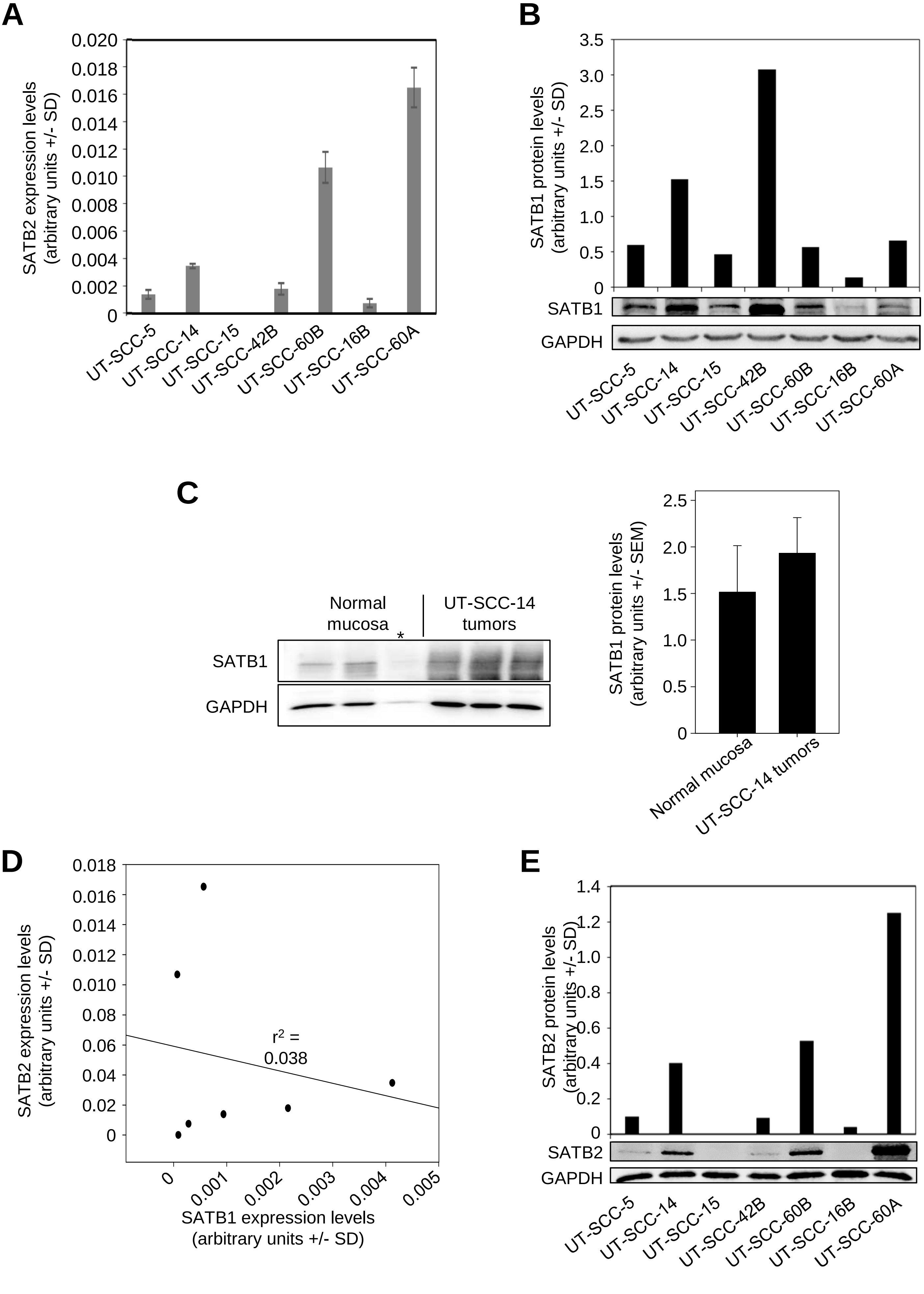

A
SATB2 expression levels
(arbitrary units +/- SD)
0.020
0.018
0.016
0.014
0.012
0.010
0.008
0.006
0.004
0.002
0
UT-SCC-5
UT-SCC-14
UT-SCC-15
UT-SCC-42B
UT-SCC-60B
UT-SCC-16B
UT-SCC-60A
B
SATB1 protein levels
(arbitrary units +/- SD)
3.5
3.0
2.5
2.0
1.5
1.0
0.5
0
SATB1
GAPDH
UT-SCC-5
UT-SCC-14
UT-SCC-15
UT-SCC-42B
UT-SCC-60B
UT-SCC-16B
UT-SCC-60A
C
Normal
mucosa
*
UT-SCC-14
tumors
SATB1
GAPDH
SATB1 protein levels
(arbitrary units +/- SEM)
2.5
2.0
1.5
1.0
0.5
0
Normal mucosa
UT-SCC-14 tumors
D
SATB2 expression levels
(arbitrary units +/- SD)
0.018
0.016
0.014
0.012
0.010
0.08
0.06
0.04
0.02
0
r² =
0.038
0
0.001
0.002
0.003
0.004
0.005
SATB1 expression levels
(arbitrary units +/- SD)
E
SATB2 protein levels
(arbitrary units +/- SD)
1.4
1.2
1.0
0.8
0.6
0.4
0.2
0
SATB2
GAPDH
UT-SCC-5
UT-SCC-14
UT-SCC-15
UT-SCC-42B
UT-SCC-60B
UT-SCC-16B
UT-SCC-60A

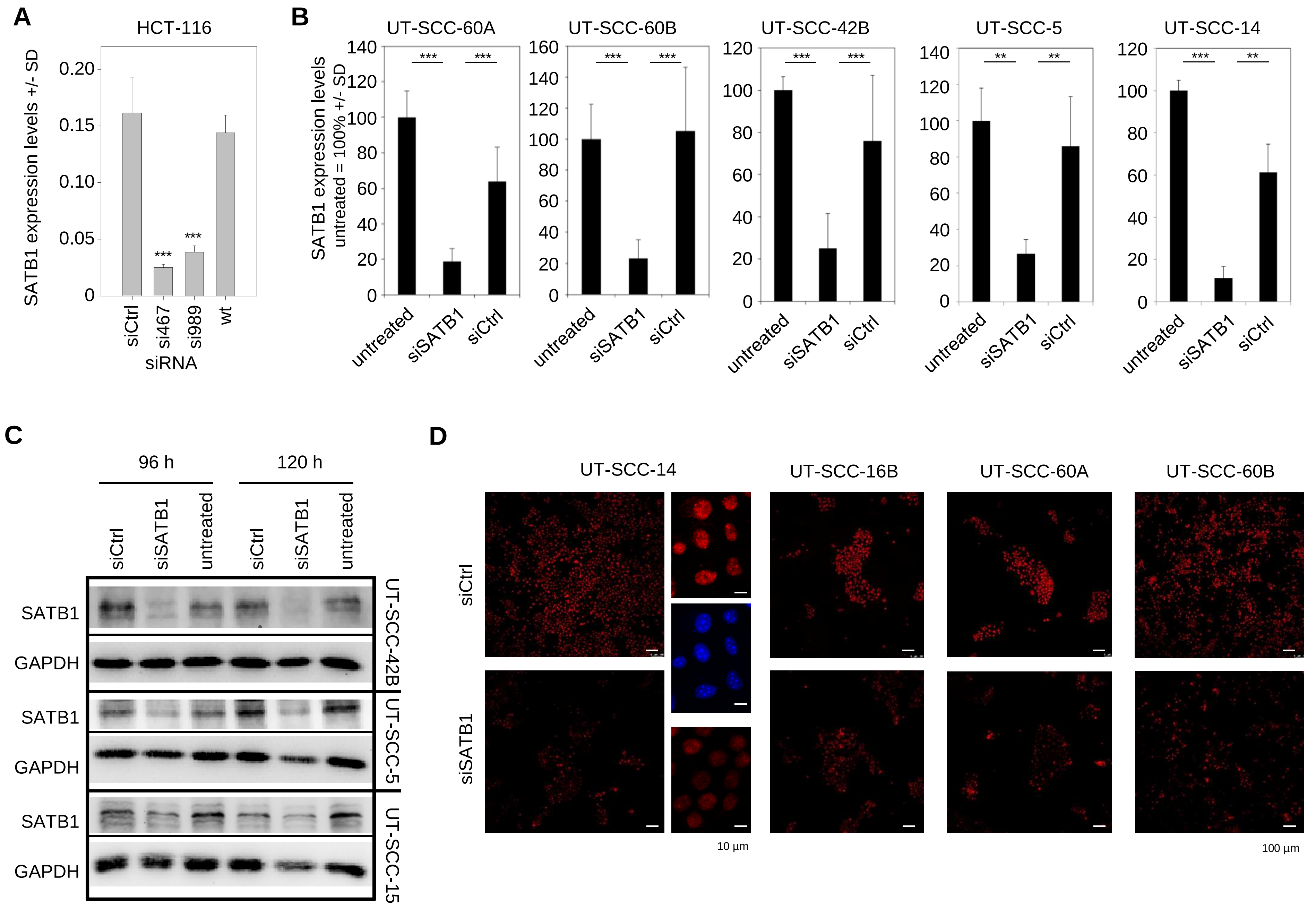

Panchal et al. – Suppl. Figure 2

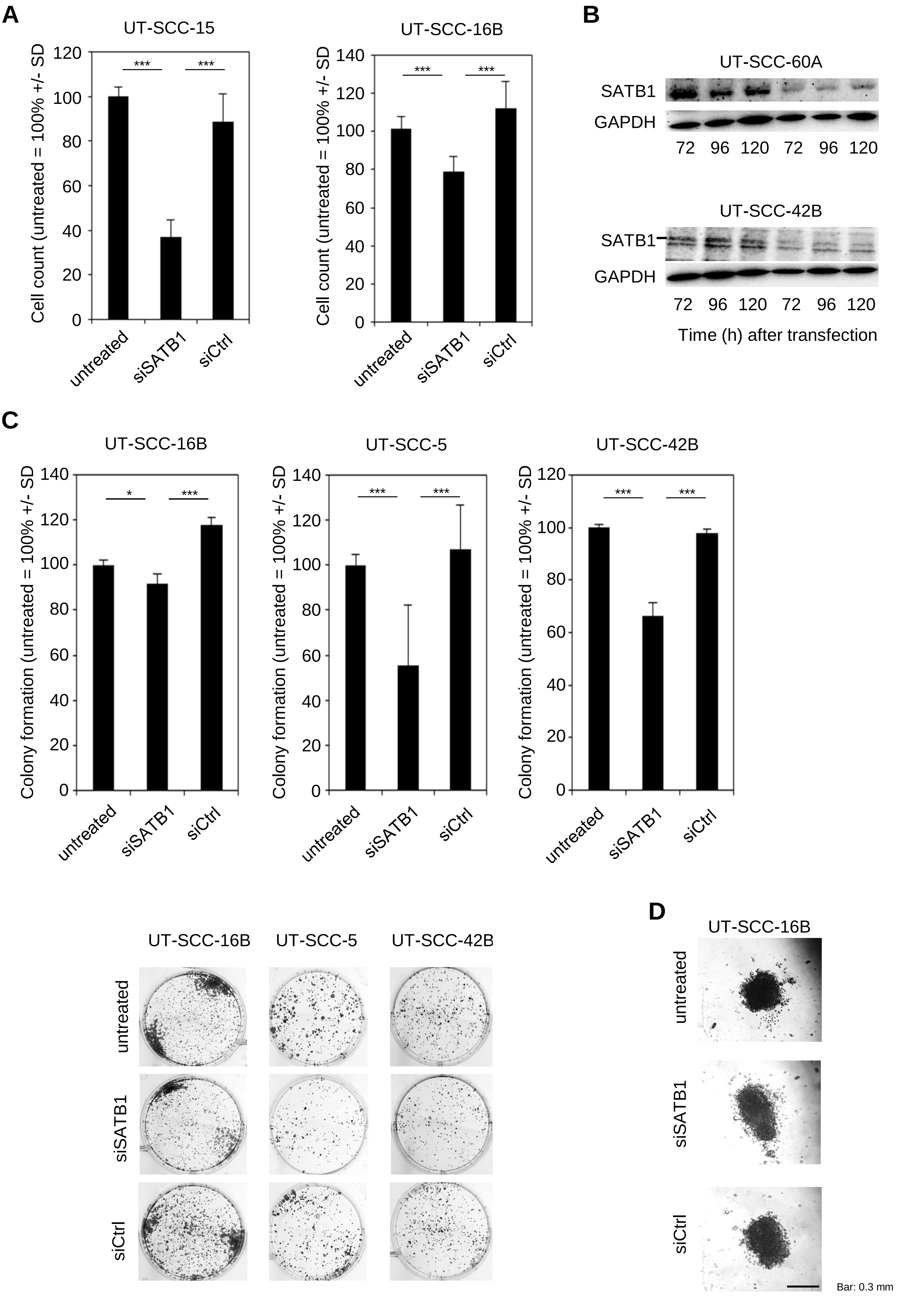

Panchal et al. – Suppl. Figure 3

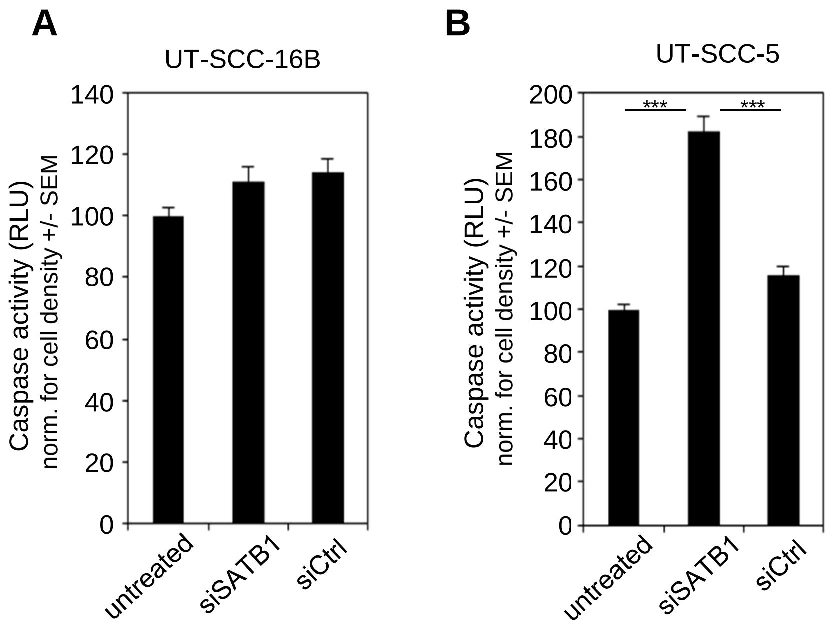

Panchal et al. – Suppl. Figure 4

A

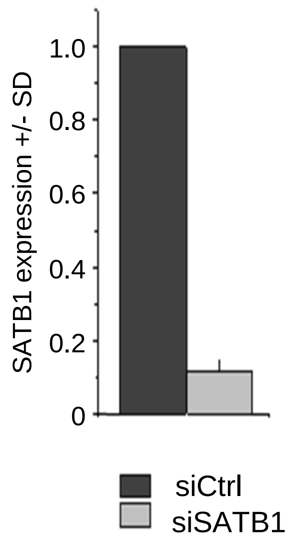

B

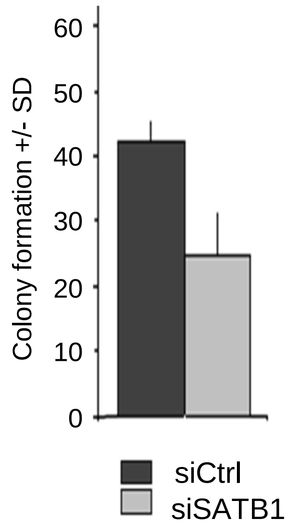

C

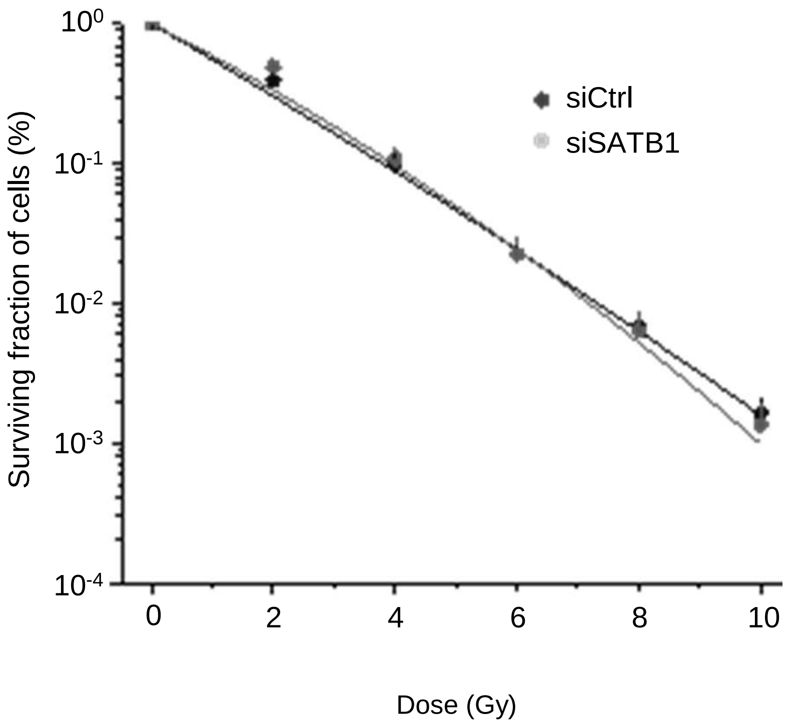

Panchal et al. – Suppl. Figure 5

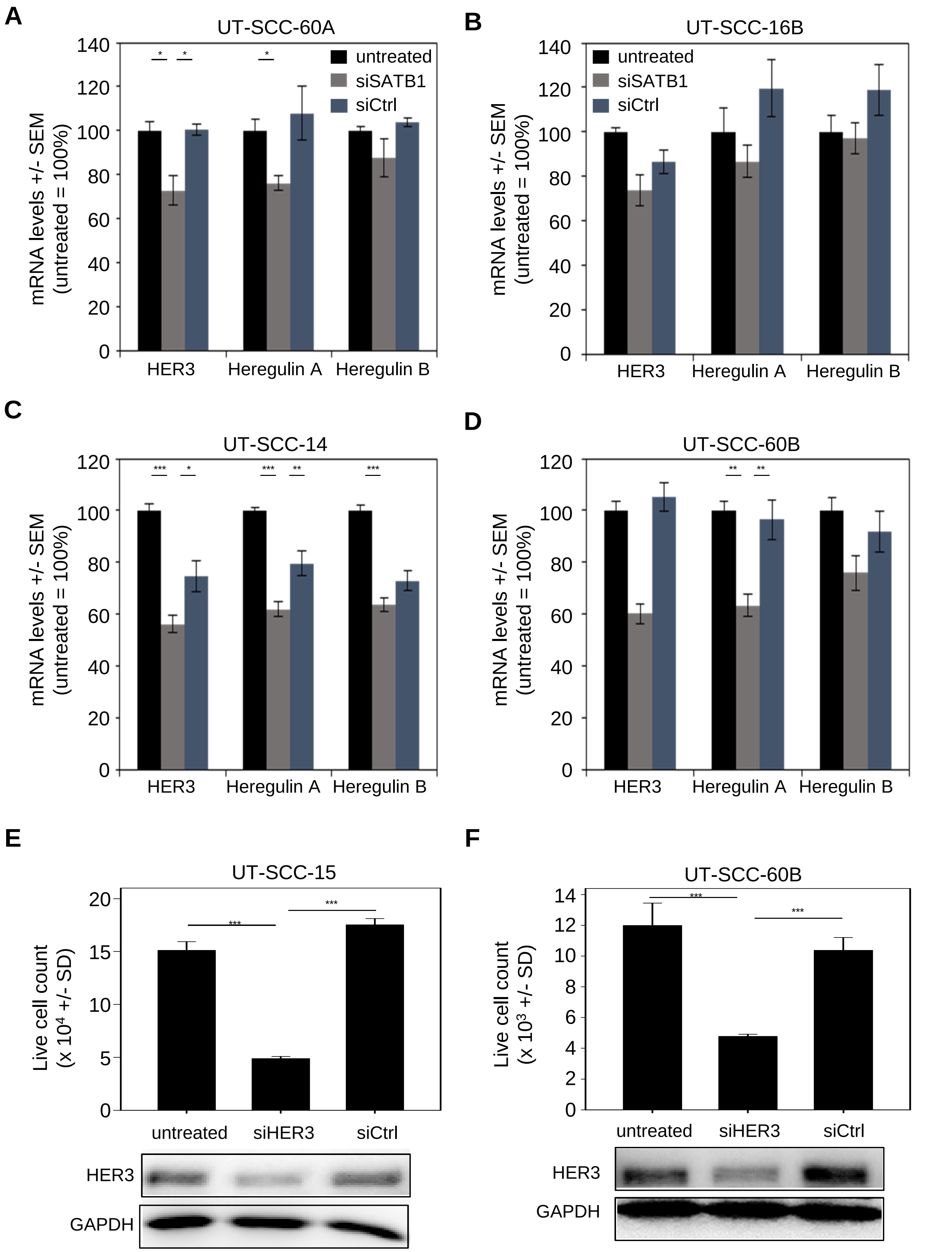

Panchal et al. – Suppl. Figure 6

A

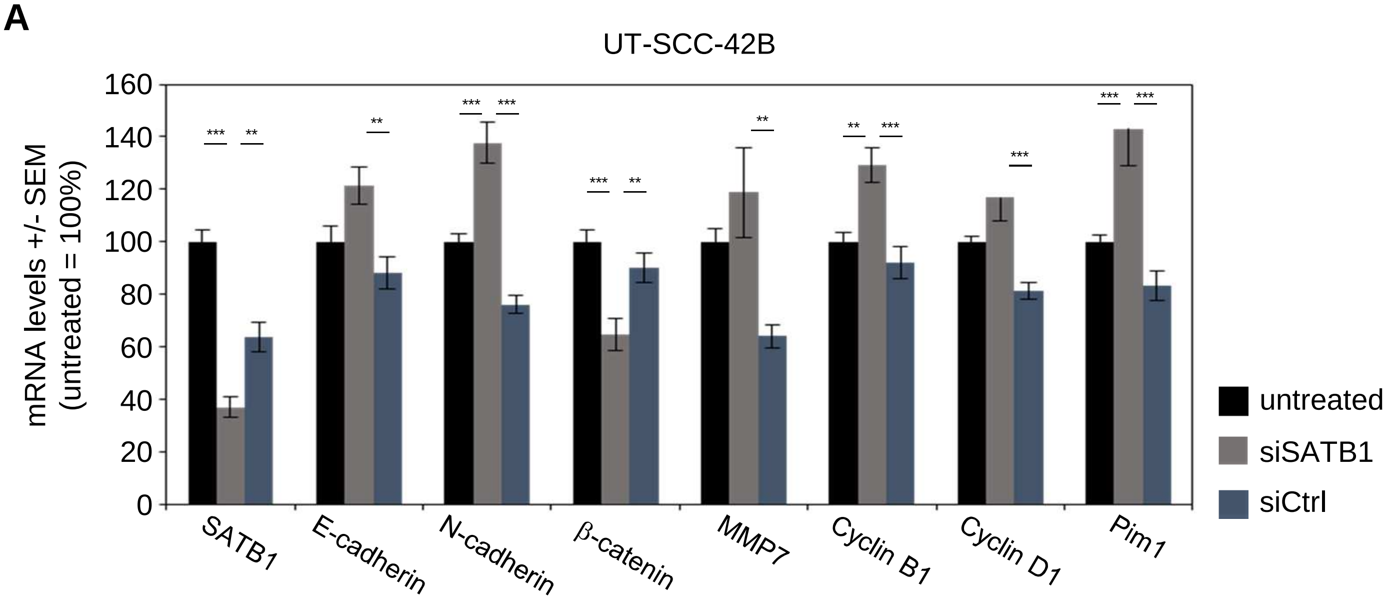

B

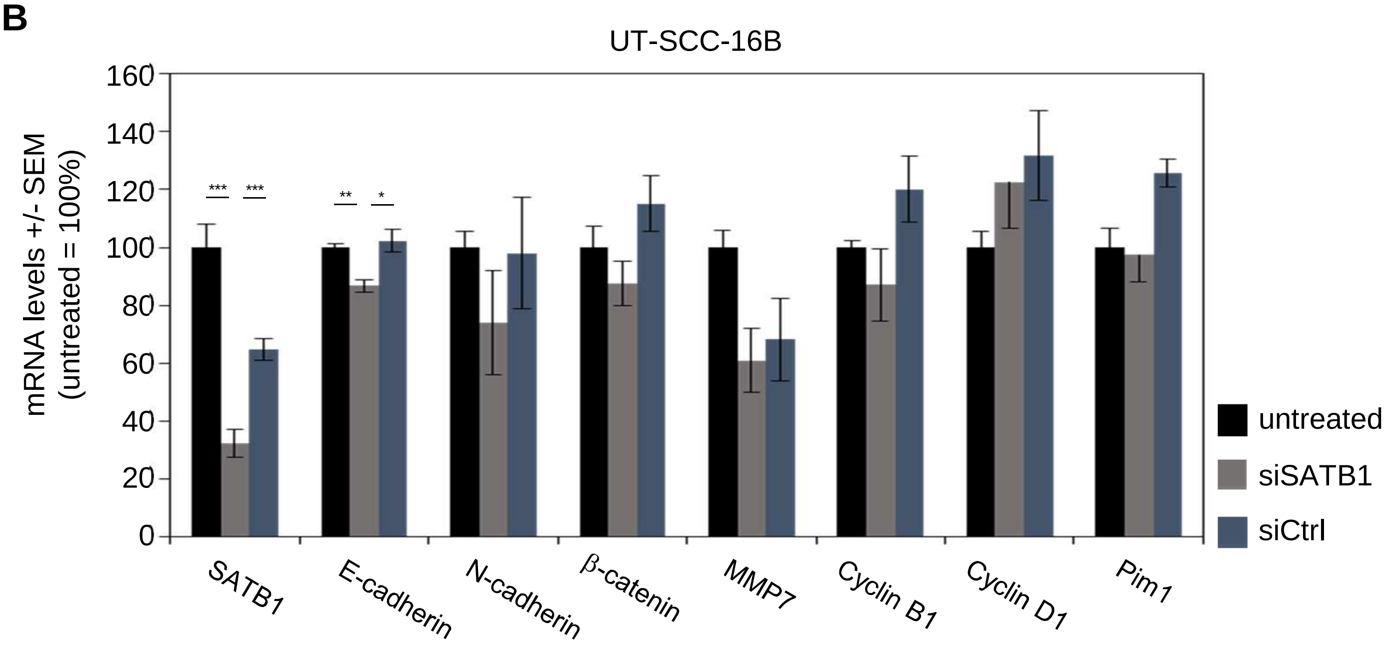

Panchal et al. – Suppl. Figure 7

Panchal et al., Suppl. Table 1

Primary cell culture	Sex	Age	Primary tumour location	TNM	Specimen site	Type of Lesion	Grade
UT-SCC-5	M	58	Linguae	T1N1M0	Tongue	Primary	G2
UT-SCC-14	M	25	SCC linguae	T3N1M0	Tongue	Primary	G2
UT-SCC-15	M	51	SCC linguae	T1N0M0	Tongue	Recurrent	G1
UT-SCC-16A	F	77	SCC linguae	T3N0M0	Tongue	Primary	G3
UT-SCC-16B	F	77	SCC linguae	T3N0M0	Neck	Metastatic	G3
UT-SCC-19A	M	44	Glottic Larynx	T4N0M0	Larynx	Primary	G2
UT-SCC-24A	M	41	SCC linguae	T2N0M0	Tongue	Primary	G2
UT-SCC-26A	M	60	SCC hypopharynx	T1N2M0	Neck	Metastatic	G2
UT-SCC-26B	M	60	SCC hypopharynx	--	Neck	Metastatic	G2
UT-SCC-31	M	58	Floor of the mouth	T3N2bM0	Floor of the mouth	Primary	G1(G2)
UT-SCC-42A	M	43	Supraglottic Larynx	T4N3M0	Larynx	Primary	G3
UT-SCC-42B	M	43	Supraglottic larynx	T3N0M0	Neck	Primary	G3
UT-SCC-60A	M	59	Left tonsilla	T4N1M0	Tonsil (left)	Primary	G1
UT-SCC-60B	M	59	Left tonsilla	T4N1M0	Neck	Metastatic (per)	G1
UT-SCC-62	M	56	Hypopharynx	T4N0M0	Neck	Metastatic	G2
UT-SCC-74A	M	31	SCC linguae	T3N1M0	Tongue	Primary	G1-G2
UT-SCC-74B	M		SCC linguae met colli	rN2	Neck	Metastatic	G2

Panchal et al., Suppl. Table 2

q-RT-PCR primers		
**cDNA**	**Orientation**	**Sequence (5'-3')**
SATB1	Forward	CGA TGA ACT GAA ACG AGC AG
	Reverse	CGG AGG ATT TCT GAA AGC AA
E-Cadherin	Forward	GGC GGA GAA GAG GAC CAG GAC TT
	Reverse	CAT CGG GAT TGG CAG GGC GG
N-Cadherin	Forward	AAC TGG GCC AGG AGC TGA CCA
	Reverse	GTG CCC TCA AAT GAA ACC GGG CT
Beta catenin	Forward	TCG AGG ACG GTC GGA CTC CC
	Reverse	CTG CCA GTG ACT AAC AGC CGC
Cyclin B1	Forward	CAT GGT GCA CTT TCC TCC TT
	Reverse	AGG TAA TGT TGT AGA GTT GGT GTC
Cyclin D1	Forward	CCT CGG TGT CCT ACT TCA AA
	Reverse	CAC TTC TGT TCC TCG CAG A
MMP7	Forward	GGG GCA AAG AGA TCC CCC TGC AT
	Reverse	CCC CAT GAG CTC CTC GCG CAA A
Pim-1	Forward	ATC AGG GGC CAG GTT TTC T
	Reverse	GGG CCA AGC ACC ATC TAA T
Survivin	Forward	TGATGAGAGAATGGAGACAGAG
	Reverse	ACAGCAGTGGCAAAAGGAG
MACC1	Forward	TTC TTT TGA TTC CTC CGG TGA
	Reverse	ACT CTG ATG GGC ATG TG CTG
HER1	Forward	ACA CAG AAT CTA TAC CCA CCA GAG T
	Reverse	ATC AAC TCC CAA ACG GTC AC
HER2	Forward	TGG CTC AGT GAC CTG TTT TG
	Reverse	GGT CCT TAT AGT GGG CAC AGG
HER3	Forward	CTG ATC ACC GGC CTC AAT
	Reverse	GGA AGA CAT TGA GCT TCT CTG G
Heregulin A	Forward	CCC ATG AAA GTC CAA AAC CA
	Reverse	CCG GTT ATG GTC AGC ACT CT
Heregulin B	Forward	GAT CAG CAA ATT AGG AAA TGA CA
	Reverse	GGC ATA CCA GTG ATG ATC TCG

Panchal et al., Suppl. Table 3: Background information on antibodies used in this study

Method	Protein	Clone/ antibody	Dilution (Diluent)	Company
Western blot	SATB1	14/SATB1	1:1000 (5% BSA in TBST)	BD bioscience
Western blot	SATB2	ab92446	1:1000 (in Pierce blocking solution)	Abcam
Western blot	GAPDH	ab 181602	1:10,000 (Pierce blocking solution)	Abcam
Western blot	Cyclin B1	ab32053	1:1000 (Pierce blocking solution)	Abcam
Western blot	HER3	ab 32121	1:1000 (Pierce blocking solution)	Abcam
Western blot	Actin	# 4970	1:2000 (Pierce blocking solution)	Cell Signaling
Western blot	Anti-mouse	Product # 31430	1:2000 (3% milk in TBST)	Thermo Fisher Scientific
Western blot	Anti-rabbit	Ab 6721	1:5000 (3% milk in TBST)	Abcam
Western blot	Anti-rabbit	827-08365	1:10,000 (Pierce blocking solution)	LI-COR
Western blot	Anti-mouse	926-68170	1:10,000 (Pierce blocking solution)	LI-COR
Immunocytochemistry	SATB1	EPR 3951	1:200 (1% BSA in PBST)	Abcam
Immunocytochemistry	Anti-rabbit (Cy3)	111-165-144	1:800 (1% BSA in PBS)	Jackson Immuno-Research
Immunocytochemistry	Hoechst 33342	H1399	1:100 (PBST)	Invitrogen
Immunohistochemistry	Ki-67	MIB-1	1:100 (2% BSA in PBST)	DAKO
Immunohistochemistry	Cleaved caspase-3	Rabbit polyclonal	1:50 (2% BSA in PBST)	MBL International
